# A Novel Digital Nutrition Diary for Geriatric Patients at High Risk of Frailty Syndrome

**DOI:** 10.3390/nu14030400

**Published:** 2022-01-18

**Authors:** Patrick Elfert, Julia Berndt, Louisa Dierkes, Marco Eichelberg, Norbert Rösch, Andreas Hein, Rebecca Diekmann

**Affiliations:** 1 R&D Division Health, OFFIS Institute for Information Technology, 26121 Oldenburg, Germany; eichelberg@offis.de; 2 Assistance Systems and Medical Device Technology, Department of Health Services Research, Carl von Ossietzky University of Oldenburg, 26111 Oldenburg, Germany; julia.berndt@uol.de (J.B.); louisa.dierkes@uol.de (L.D.); andreas.hein@uol.de (A.H.); rebecca.diekmann@uol.de (R.D.); 3 Department of Computer Sciences and Microsystems Technology, University of Applied Sciences Kaiserslautern, 66482 Zweibrücken, Germany; norbert.roesch@hs-kl.de

**Keywords:** digital nutrition diary, nutrition counseling, user-centered design, frailty syndrome, geriatric rehabilitation, system usability scale

## Abstract

Due to the physical, psychological, or socioeconomic changes that accompany aging, many people will be affected by geriatric frailty syndrome, which can lead to multimorbidity and premature death. Nutrition counseling is often used to prevent and intervene in frailty syndrome, especially in geriatric rehabilitation. To this end, the consumption behavior of geriatric patients is recorded using paper-based, as well as retrospective memory logs in face-to-face interviews between patients and nutritionists. To simplify this procedure, a digital nutrition diary was developed that is specially adapted to the needs of geriatric patients (>=70 years), enabling them to record their consumption behavior themselves. In an initial study (Study 1), conducted in a geriatric rehabilitation division with twelve subjects (ten male, two female, mean age 79.2 ±5.9 years), feedback about the usability of the digital nutrition diary, and how to improve it, was surveyed. In addition, the usability of an activity tracker and a body composition scale was surveyed to determine whether geriatric patients are generally able to use these devices. In a second study (Study 2), also conducted in the geriatric rehabilitation division, this time with sixteen subjects (ten male, six female, mean age 79.3 ±3.9 years), the usability of the digital nutrition diary was surveyed again to evaluate its modifications based on the feedback from Study 1. In Study 1, the usability rating of the system (0–100) was 82.5 for the activity tracker, 29.71 for the body composition scale, and 51.66 initially for the digital nutrition diary, which increased to 76.41 in Study 2.

## 1. Introduction

Most industrialized societies are currently undergoing a process referred to as ″demographic change″. While in 2004, there were about 461 million people over the age of 65 throughout the world, this number is estimated to rise to 1.5 billion by 2050 [1,2]. Since this section of the population will be affected more frequently by diseases and health impairments, this increase in those over 65 will pose immense challenges for both medical and social care.

To be precise, geriatric frailty syndrome, which is expected to affect 25% to 50% of all people over 85 years of age [3] and is often associated with malnutrition [4], should not be underestimated. The DiDiER project [5] addressed this issue by exploring how to establish digitalized nutrition counseling services for groups of people at an increased risk of malnutrition. One of the project’s use cases therefore focused specifically on supporting nutrition counseling for people affected by frailty syndrome. The goal was to improve the quality and the quantity of data for nutrition counseling, while reducing the workload of traditional retrospective and paper-based data acquisition methods for nutritionists and patients. The content described in this article is based on work conducted in the context of the DiDiER project [6].

### 1.1. Frailty Syndrome

Frailty is a multidimensional and multifactorial geriatric syndrome triggered by the physiological aging process and its pathological consequences [4,7,8,9]. Although no clear definition of the syndrome exists as of yet, it is generally agreed that frailty is a physiological condition associated with reduced physiological reserves and increased vulnerability to adverse health events and external stressors [10]. While it is normal for the body’s physiological reserves to slowly decline in later life, this decline is accelerated in geriatric frailty, and homeostatic mechanisms begin to fail [11,12]. Older people affected by frailty syndrome are at a significantly higher risk of falls, fractures, infections, hospitalizations, multimorbidity, and premature death than older people who are not affected by the syndrome [13,14,15,16,17,18,19].

A commonly used and widely accepted model for the syndrome is the frailty phenotype according to Fried et al. [4]. In this model, the main indicators for the presence of frailty syndrome are a reduced handgrip strength, walking speed, physical activity, and body weight and increased fatigue. If three or more of these indicators are present, a person is considered to be frail. People with one or two indicators are classified as pre-frail, and people affected by none of these indicators are described as robust [4,9].

### 1.2. Nutrition Counseling

As previously described, frailty syndrome is mainly characterized by a loss of muscle mass, muscle strength, and general physical activity. Consequently, physical rehabilitation is both an obvious treatment and a preventive measure. Different studies have shown that these physical rehabilitation measures work well when combined with an appropriate therapeutic diet [20,21,22]. Furthermore, evidence exists that an inadequate nutritional intake is also an independent and modifiable risk factor for the prevention and treatment of frailty [23]. Moreover, epidemiological studies have described associations between various nutritional parameters and frailty, e.g., malnutrition and the risk of malnutrition, reduced body weight, and reduced dietary quantity and quality [23,24,25,26]. In terms of dietary quantity, the intake of adequate energy, protein, and micronutrients is particularly low in individuals with frailty syndrome [24,26,27].

In addition to total caloric energy, the amount of protein supplied is particularly important, since this macronutrient is known to be an essential material for building and maintaining muscle mass and strength. However, a lack of other macronutrients, such as fats or carbohydrates, also leads to an approximately 10% higher risk of becoming frail [28]. The problem is that many geriatric patients are not aware of these associations. Since many patients do not realize that a loss of body weight in later life is usually accompanied by a considerable loss of muscle mass, such weight loss is often not perceived as a problem, but as something positive, e.g., due to the presence of obesity.

Particularly in the geriatric context, nutrition counseling and the acquisition of data often take place in face-to-face meetings between the nutritionist and the patient [29]. Typically, paper-based questionnaires are used to record data about the patient’s nutritional status, e.g., using the Mini Nutritional Assessment (MNA) [30] or the Mini Nutritional Assessment Short-Form (MNA-SF) [31], or about their consumption behavior, e.g., using the 24 h recall protocol [32]. Besides providing a single assessment of the patient’s current status, these protocols also enable the nutritionist to assess the course of a therapeutic diet through repeated use.

However, retrospective and memory-based protocols require patients to perform at a high cognitive level. In particular, the 24 h recall protocol requires patients to remember in detail all foods, beverages, and dietary supplements they have consumed within the past 24 h. It is not only important which individual foods, beverages, and dietary supplements were consumed during the various main meals, but also whether they had any snacks in between. Snacks, in particular, can vary widely and are not always available, so they can quickly be forgotten or incompletely reported when a person tries to remember the exact number of cookies they ate, for example. Even if patients are able to remember all the foods, beverages, and dietary supplements they consumed, and then accurately report them in terms of quantity, nutritionists will only obtain information about the consumption behavior of their patients for a single day.

Another method is the plate diagram sheet (PDS), which was developed to allow the semi-quantitative recording of consumption quantities by patients. The aim was to develop a method that can be integrated into the clinical routine with minimal effort. The PDS is used to estimate the portion of a meal consumed in 25% increments, e.g., recorded by the nursing staff of the respective facility. One significant benefit of the PDS is that it can be easily integrated into existing patient documentation [33]. Since the PDS is a quantitative estimation of consumption quantities, there is no distinction whether a patient has eaten half of his/her meal with vegetables, for example, or the other half with high-fat meat, for instance. This can lead to corresponding differences between the estimated volume and the actually consumed calories, micronutrients, and macronutrients [34]. However, when using the PDS, it is important to keep in mind that it is typically administered by nursing staff at an appropriate facility at regular meal times and that it does not consider snacks between meals. Even if PDS-based estimations are considered sufficient for individual use cases, the resulting data are usually only available in paper-based form. A subsequent manual digitalization can be an additional source of error.

### 1.3. Digital Nutrition Acquisition

In addition to recording the intake of foods, beverages, and dietary supplements with paper-based diaries, research has been ongoing for decades to determine how and to what extent computerized systems can be used to support this process [35,36]. Nutrition calculation programs (e.g., DGExpert [5]) have become indispensable, especially for nutritionists [37,38]. Another example of the digital recording of foods, beverages, and dietary supplements is the Diet Interview Software for Health Examination Studies (DISHES 98) software. DISHES 98 was implemented as a modified dietary history interview, which allows trained nutritionists to directly record consumption behavior digitally during a face-to-face interview. The resulting data are linked to a food database, providing immediate results for calculated caloric, as well as macronutrient and micronutrient intake [39]. Another example is the Prospective Investigation into Cancer and Nutrition (EPIC-SOFT) system, which was developed based on the 24 h recall protocol. The software was originally used by trained individuals to digitally record nutrient intake directly during telephone interviews. In addition, the system supports the interview process in order to make it as consistent as possible and to minimize variations between interviewers [40].

In addition to expert tools, the development of smartphones for the mass market has led to the creation of mobile applications that allow users to self-report their consumption behavior. In addition to consumer market applications for people who record their intake of foods, beverages, and dietary supplements out of self-interest, without any nutrition counseling, there are also applications that connect people with nutritionists. Examples of widely used self-tracking applications in the consumer market include YAZIO [41], MyFitnessPal [42], and Lifesum [43]. These apps enable users to independently log their consumption behavior and often also other data about their body weight or physical activity. Food, beverage, and dietary supplements in particular are usually based on a manual textual search in a database linked to the application. Since these databases are very extensive, the app usually needs to be connected to the Internet. OVIVA, on the other hand, is an example of a platform that brings together nutritionists and patients to provide support, e.g., for obesity, food allergies and intolerances, or malnutrition. Therefore, OVIVA [44] was developed for a wide range of target groups [45,46]. Even though, or because, these applications are designed for a wide range of users and patients, they are usually not adapted to the specific needs of older people. Since these needs, such as large icons and fonts, are essential for their effective use and associated acceptance, this target group usually requires specially adapted software systems [47,48].

### 1.4. Objective

The aim of this study was to evaluate whether geriatric patients are able to independently log their consumption behavior with the help of a digital nutrition diary (DND). Furthermore, it was evaluated whether off-the-shelf sensor technology, specifically selected according to the needs of the target group, can be used by geriatric patients to log data about their physical activity (e.g., steps) and body composition (e.g., body weight or fat-free mass).

A major challenge has been that many geriatric patients have little or no experience at all with mobile applications, sensors, or similar technologies. For this reason, the focus in the development of the DND and the selection of the sensor technology was on optimal usability for the target group of geriatric patients. To this end, an initial version of a digital nutrition diary (DNDv1) was developed in close collaboration between experienced nutrition counseling experts and developers. The sensors, an activity tracker (AT) and a body composition scale (BCS), were also selected in this collaboration. To evaluate the selection of the AT, the BCS, and the development of the DNDv1 in terms of usability, Study 1 was conducted; see Section 2.2. In addition, data and feedback were acquired in Study 1 to further adapt DNDv1 to the needs of geriatric patients and thus improve its usability. The resulting second version of DNDv2 was evaluated in Study 2; see Section 2.3.

## 2. Materials and Methods

For DNDv1 and for the selection of the AT and the BCS, the associated requirements were developed in close collaboration between experienced nutritionists and developers. To this end, various personas of geriatric patients and nutritionists were created, describing the present state of the use case without digitalization in so-called user stories. The aim was to identify needs and then convert them into requirements. With the help of these requirements, new user stories were developed for the target state, describing the use case, including digitalization. The personas, the present-state user stories, and the target-state user stories were developed iteratively between nutritionists and developers to represent real-world user stories and the resulting requirements as accurately and circumferentially as possible. This approach was chosen to involve the target group of geriatric patients at the later state of the development process, in Study 1, to avoid burdening them with early prototype testing.

For geriatric patients, it is important that the cognitive and sensomotoric requirements for operating DNDv1, the AT, and the BCS are kept to a minimum. As described above, the AT and the BCS were not developed within the project. These two devices were selected from the wide range of existing products. The smart body scale Withings^®^ Body Cardio [49,50] was chosen to measure body composition data because of its flat design, large standing base, and large illuminated display. The Withings^®^ Activité Pop [51] smart watch was selected to measure activity because of its similarity to a conventional wristwatch in terms of handling and shape. Moreover, the Withings^®^ Activité Pop does not require daily or weekly charging.

### 2.1. Digital Nutrition Diary

**Figure 1:**
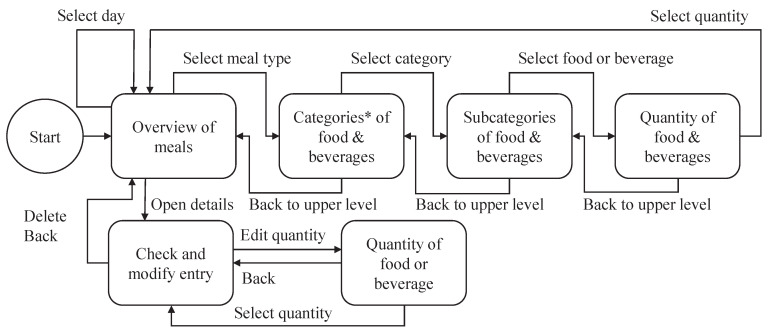
Overview of the architecture of DNDv1, including all possible navigation steps for recording foods and beverages. Notes: (*) For DNDv2, two additional categories were added: the ″Most used″ and the ″Supplements″ categories. Furthermore, the selection of the day is limited to today, yesterday, and tomorrow. DNDv1, digital nutrition diary version 1; DNDv2, digital nutrition diary version 2.

**Figure 2:**
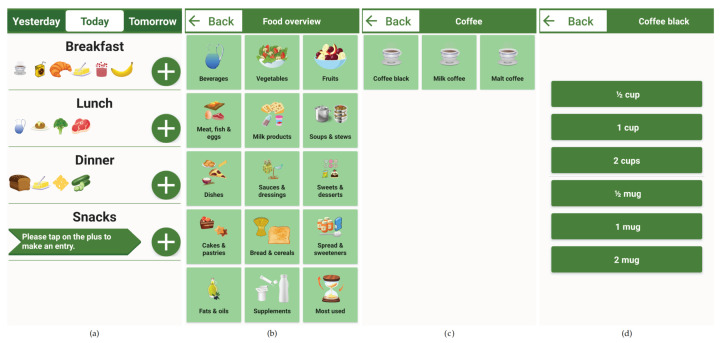
Partially completed nutrition diary for a single day (**a**), including the navigation steps for selecting ″Coffee black″ (**b**,**c**) and the corresponding household quantity (**d**). The recording is started by clicking on the ″Plus″ button of a specific meal (**a**). Adding a cup or a mug of coffee to DNDv1 took five navigation steps in this case. Note: All screenshots were generated based on DNDv2. Therefore, the selection of the day only allows yesterday, today, or tomorrow and the two categories ″Most used″ and ″Supplements″ can be seen. Furthermore, the subcategory overview ″Coffee″ was omitted for clarity, and the original application evaluated in German was translated into English. DNDv1, digital nutrition diary version 1; DNDv2, digital nutrition diary version 2.

As mentioned earlier, the priority during the development of DNDv1, and also during the subsequent development of DNDv2, was to make the recording of consumption behavior as intuitive as possible for geriatric patients. A complex and nested structure was therefore avoided, and a navigation structure with maximum linearity was developed, as shown in Figure 1. Furthermore, factors such as simple language and content visualization by means of pictographs were taken into account; see Figure 2.

After starting DNDv1, the daily overview of meals screen is displayed. In this view, the recording of foods and beverages can be started by clicking on the corresponding ″Plus″ button. As long as no entry has been made for a corresponding meal (breakfast, lunch, dinner, or snacks), there is also a note in an arrow-shaped text box for the ″Plus″ button; see Figure 2. Then, the overview of the main food categories is displayed. Here, both the corresponding pictograph and the associated textual description can be clicked to access a specific subcategory. This can be repeated until the desired food or beverage is reached. The data structure in which these foods and beverages and dietary supplements—included later in DNDv2—are inserted is called the foods, beverages, and dietary supplements tree (FoBaST). A leaf of this tree, e.g., a single food, beverage, or dietary supplement, in DNDv2 is called a FoBaST item. After selecting a FoBaST item, the user is automatically directed to the quantity selection screen.

Nutrition diaries often work with precise quantities specified in grams or milliliters. However, the practice of nutrition counseling has revealed that many patients have problems with this type of quantity reporting, because they are generally unable to estimate quantities accurately, and an appropriate scale is often not available. For this reason, common household quantities were used, such as ″cups″ or ″mugs″ for some types of beverages, ″slices″ for bread and for cold cuts, or ″handful″ for fruit and vegetables; see Figure 2a. To avoid the use of the on-screen keyboard, buttons were integrated for scaling these common household quantities; see Figure 2d. Up to this point, the user is able to navigate back each step via a ″Back″ button. However, if the user now selects one of the household quantities, the daily overview of meals screen is displayed where the corresponding FoBaST item is now added; see Figure 2a. By clicking a FoBaST item that has been entered in the daily overview of meals screen, the user can view the macronutrients and calories based on the selected quantity, edit the quantity, or delete the entry. The user can also view the FoBaST items entered on previous days to make additional entries or corrections. Moreover, the user can make entries for the following days if the FoBaST items and quantities have already been determined for those days.

The DND logs every user interaction in the background, allowing for the evaluation and appropriate optimization of the design. Furthermore, this logging should allow for a better understanding of any problems that geriatric patients may have with the navigation elements of DNDv1. Moreover, DNDv1 was designed to permit installation on a mobile device such that the app is automatically started and displayed in the foreground when the device is switched on. Should the device restart, due to an empty battery or pressing the on/off button for too long, the user is automatically returned to the daily overview of meals screen.

### 2.2. Study 1

The participants’ task in this study was to record a single meal—lunch—on a single day using DNDv1, which was preinstalled on a 10 in tablet computer and provided to the participants. Initially, the participants were given an introduction to DNDv1, which lasted around 25 min and included a sample recording of a complete meal. Furthermore, the participants were to use the AT for 90 min during a physiotherapeutic treatment and the BCS to measure their body weight. The participants’ technology commitment was assessed using the Technology Commitment (TC) [52] questionnaire. The usability of DNDv1, the AT, and the BCS was assessed separately using the System Usability Scale (SUS) [53] questionnaire; see Section 2.5.

### 2.3. Study 2

The participants’ tasks in Study 2 were more extensive compared to Study 1. They had to record all foods, beverages, and dietary supplements consumed over a total of three days for all meals (breakfast, lunch, dinner, and snacks) with the help of DNDv2. These three days had to be consecutive and had to include one weekend day and two working days. As in Study 1, the TC and the SUS questionnaire were surveyed; see Section 2.5.

### 2.4. Participants

Inclusion criteria for geriatric patients to participate in both studies were a minimum age of 70 y and an in-patient admission to a geriatric rehabilitation division. Exclusion criteria for both studies were a lack of capacity to give consent, insufficient capacity to understand the study content and the procedure, or participation in other studies. Furthermore, insufficient mobility, e.g., being bedridden, led to the patients’ exclusion, because they would not have been able to use the AT and the BCS.

The frailty syndrome or frailty phenotype was not surveyed, since the system as a whole is intended to be suitable for geriatric patients so that it can also be used preventively. Furthermore, the authors know from previous studies that the geriatric patients at the institution where the studies were conducted consisted of 63.8% frail patients, 33.6% pre-frail patients, and 2.5% robust patients according to the frailty phenotype, based on a survey with a total of 122 geriatric patients [54].

### 2.5. Questionnaires

Two questionnaires were used in both studies; the System Usability Scale (SUS) [53] and the Technology Commitment (TC) questionnaire [52]. The SUS contains five questions on the usability of a software system. There questions are formulated once positively and once negatively, resulting in a total of ten questions. The answers to the questions are given on a five-point scale, ranging from ″Strongly disagree″ to ″Strongly agree″. For the positively worded questions, the value of the scale minus one is added to the score. For the negative answers, five minus the value of the scale is added to the score. The result is multiplied by 2.5 to give a range of 0–100, were 0 is the worst and 100 the best score possible [55].

The TC questionnaire contains a total of 12 questions that focus on technology acceptance, technology competence, and technology control beliefs. As with the SUS, the TC questionnaire has positively and negatively worded questions that are answered with a five-point scale, ranging also from ″Strongly disagree″ to ″Strongly agree″, and negatively worded questions that need to be converted. Unlike the SUS, the result of each question is not reduced by 1. Hence, for positively worded questions, the value of the scale is directly added to the score. For the negative answers, six minus the value of the scale is added to the score. This results in a range of 12–60 points for the lowest to highest technology commitment [52,56].

Study staff were present during Study 1. They observed the participants while they used DNDv1, the AT, and the BCS. Positive and negative observations, as well as feedback from the participants were documented directly. These observations were evaluated in close collaboration among study staff, nutritionists, and developers to identify problems with the use, as well as any useful modifications and additional features. Afterwards, most of the relevant topics were integrated into DNDv2.

## 3. Results

**Figure 3:**
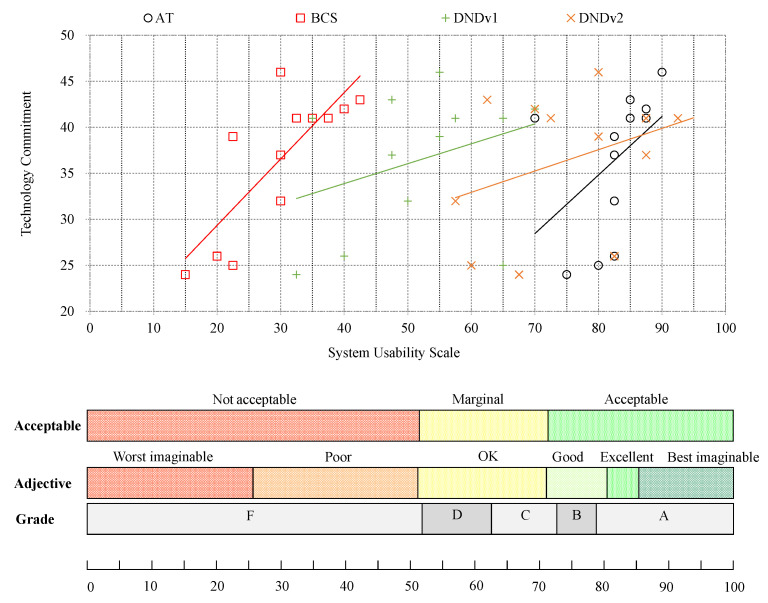
Overview of the SUS results of DNDv1 (mean SUS = 51.66), the AT (mean SUS = 82.50), and the BCS (mean SUS = 29.79) from Study 1, as well as the SUS results of DNDv2 (mean SUS = 76.40) from Study 2 in relation to conventional ranking, using grades, adjectives, and acceptance levels in correlation with the TC score. SUS, System Usability Scale; DNDv1, digital nutrition diary version 1; AT, activity tracker; BCS, body composition scale; DNDv2, digital nutrition diary version 2; TC, Technology Commitment.

In Study 1, the data of twelve participants (ten males, two females, mean age 79.2 ±5.9 years) were analyzed. Figure 3 shows the results of the TC and SUS questionnaires from Study 1 for the AT, the BCS, and DNDv1 and DNDv2 from Study 2. It also describes the development of DNDv2 according to the feedback obtained from Study 1.

The participants’ feedback for DNDv1 in Study 1 was categorized into three distinct topics: function, content, and user interface.

**Function**: With regard to the functions of DNDv1, six test persons stated that they were unsure about not having to explicitly confirm their entries. They therefore did not know whether and when their entries had been saved.
**Content**: One subject requested searching for FoBaST items via a text field. Concerning the content of DNDv1, missing FoBaST items (e.g., ketchup, mayonnaise, tartar sauce, crab sauce, mustard sauce, and curry sauce) were reported 13 times. In addition, some foods and beverages were not searched by the participants in the designated categories.
**User Interface**: The feedback on the user interface of DNDv1 was mainly related to the navigation elements, e.g., buttons that were too close to each other or buttons that could not be interpreted intuitively by the participants.

A search function via a text field was not integrated, because this may possibly have been able to help individual and technically experienced geriatric patients, but, in the opinion of the nutritionists, would have caused irritation among the majority of geriatric patients. An additional notification for the saving of each recorded FoBaST item was not implemented either, as this is done automatically and would lead to significant delays. To address the feedback regarding content, the corresponding FoBaST items were added. Furthermore, adjustments were made to the appearance, the position, and the spacing of navigation elements to provide the target group with a more intuitive and easier-to-use interface. Here, for example, the buttons for entering the quantity of an item were spaced further apart from each other, as the test participants sometimes had difficulties pressing them accurately due to motor impairments.

**Figure 4:**
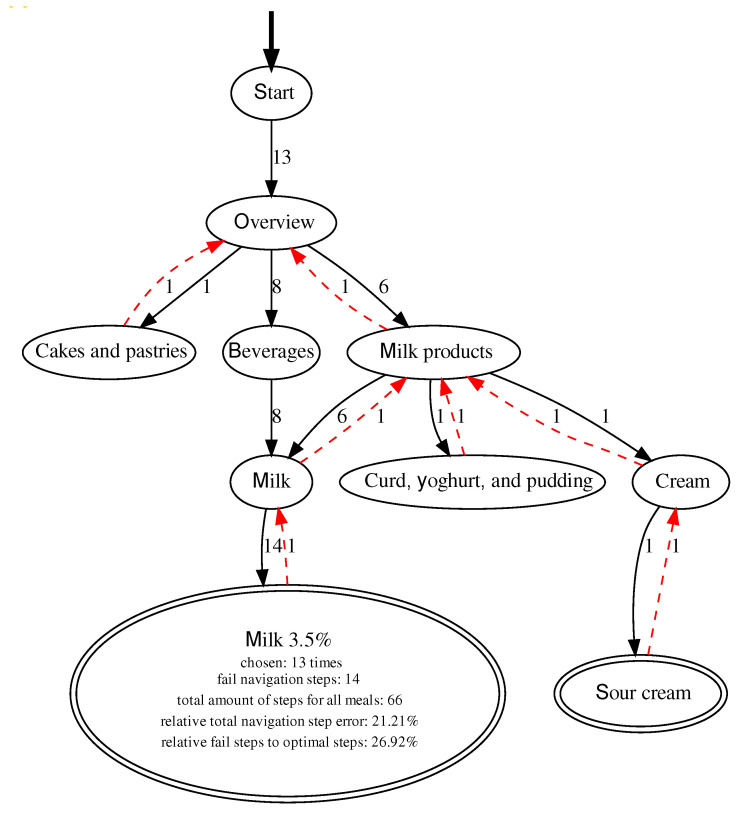
Example graph with all valid paths for the FoBaST item “Milk 3.5 %”, including all misnavigation steps. Here there are both forward navigation steps, indicated by black arrows, and backward navigation steps, indicated by red arrows. Note: Each misnavigation step includes both a forward and a backward navigation step.

Adding more items to the FoBaST makes it larger and more complex. However, since the participants already had problems finding the corresponding FoBaST items when entering just one lunch, the positioning of existing and new FoBaST items was identified as a general issue. Using the results from the analysis of DNDv1 and DNDv2 interaction logging data, it was possible to analyze the participants’ behavior with regard to navigation errors or uncertainties. However, in order to perform such an analysis, the various data records first had to be linked to each other to create valid navigation paths. A valid navigation path can be mapped to a single FoBaST item. It starts with the ″Plus″ button and ends with the selection of a household quantity. This results in one path per recorded FoBaST item. These paths can be aggregated per FoBaST item and mapped back onto the structure of the FoBaST in order to calculate navigation error rates based on the optimal path; see Figure 4. Furthermore, it was determined that a valid navigation path may not exceed an intermediate time of five minutes, based on explorative data analysis, between navigation steps. In the case of longer interruptions, it was assumed that the recording was interrupted or canceled.

The participants were not instructed to use DNDv1, or DNDv2, exclusively for logging their consumption behavior during either Study 1 or Study 2. Consequently, some participants tried out DNDv1, or DNDv2, to explore which FoBaST items were available. This could be observed especially in the resulting paths of DNDv2 in Study 2. During Study 2, the participants also used DNDv2 to record additional meals on days that did not count as study days. Such behavior resulted in chaotic paths, which were nevertheless part of the evaluation if they fulfilled the corresponding conditions for valid paths. Nevertheless, misnavigation times for special FoBaST items accumulated, e.g., milk was often searched in the categories: ″Beverages″ and ″Milk products″; see Figure 4. Other examples are yogurt and curd dishes, which were often searched in the ″Milk products″ category, as well as in the ″Sweets & desserts″ category. Consequently, against the initial assumption that it would be easier and clearer for geriatric patients if FoBaST items occurred exactly once, the corresponding FoBaST items were placed more than once in the FoBaST. However, all changes were discussed with the nutritionists for additional validation before being integrated into the FoBaST.

Close collaboration among nutritionists, study staff, and developers resulted in further opportunities to further adapt the usability to the target group. Since Study 2 involved multiple meals on multiple days, the category ″Most used″ was added to the main food categories screen to directly access frequently selected FoBaST items; see Figure 2b. This category contains all FoBaST items entered for the selected meal in descending order and updates itself with each FoBaST item entered to dynamically adapt to the user’s consumption behavior. Consequently, as soon as the first FoBaST item was recorded, the associated item would also appear in the ″Most used″ category of the associated meal. Furthermore, the ″Supplements″ category was added to the main food categories screen, containing dietary supplements commonly used in the geriatric rehabilitation division; see Figure 2b.

Based on the experience with the participants from Study 1 and the expertise of the nutritionists, it was also decided to decrease the functional scope of DNDv2 in comparison to DNDv1. The option to record consumption quantities over several days in retrospect, as well as in advance was limited to one day at a time, as the selection function for several days sometimes confused the participants.

**Figure 5:**
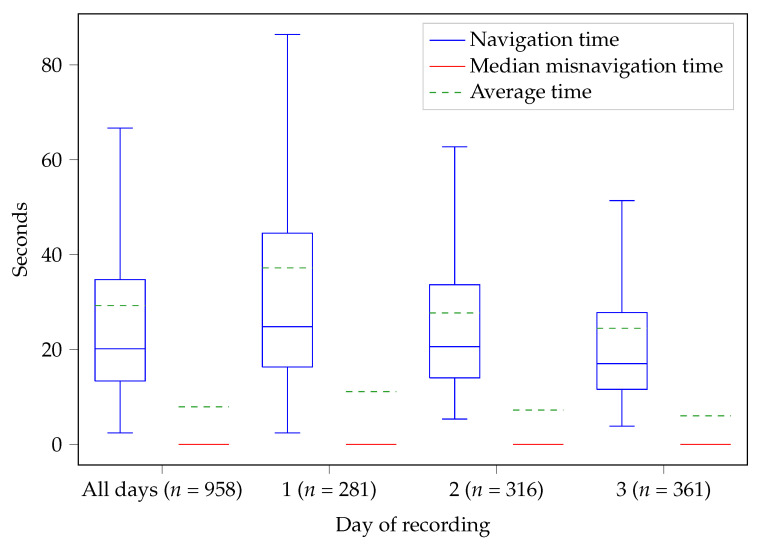
Comparison of the times needed by the participants of Study 2 to record a single FoBaST item. The mean time ($t_{mean}$) and the mean misnavigation time ($t_{mis}$) are 29.28 s and 7.93 s for all three days, 37.20 s and 11.14 s for the first day, 27.73 s and 7.25 s for the second day, and 24.49 s and 6.03 s for the third day. Note: The red lines—the median misnavigation times—are 0 because more than 50 % of the corresponding paths have no misnavigation times.

The results of the analysis of the TC and SUS questionnaires from Study 2 for DNDv2 are depicted in Figure 3. Study 2 was fully completed by a total of sixteen participants (ten males, six females, mean age 79.3 ±3.9 years). In addition, the path analysis was used to calculate how long the participants needed to enter individual FoBaST items on the different study days; see Figure 5.

## 4. Discussion

Comparing the results of the usability of DNDv1 and DNDv2, the correspondingly performed analyses, changes, extensions, and restrictions for the development of DNDv2 can be considered as successful. Furthermore, the usability of the AT in Study 1 showed that off-the-shelf sensor technologies already exist that can be used by the target group. Even though the evaluation of the AT in Study 1 focused on usability, it should be mentioned that recent studies described that the precise recognition of steps remains a challenge, especially at low speeds or when patients used a gait aid, so the precise recognition of steps remains a challenge [57,58]. In contrast to the AT, the problems experienced by the participants with the BCS revealed that the needs of geriatric patients are complex and do not necessarily match the properties of even carefully selected devices. Despite the below-average performance of the BSC in terms of usability, it can be stated that, from the perspective of usability, it can enable most geriatric patients to independently and continuously log their consumption behavior and their physical activity themselves. This can improve the data quality and quantity for nutritionists, since DNDv2 can be used by a geriatric patient immediately after a meal, avoiding significant time gaps between consumption and recording events.

For both studies, it must be taken into account that the participants used DNDv1 and DNDv2 for only a relatively short period of time. In particular, the evaluation of the average time required to record a meal revealed a learning effect over the three days of recording during Study 2 and suggested that participants would be able to use DNDv2 even more effectively following longer-term use; see Figure 5. This learning effect may also have had an impact on the SUS results. Furthermore, the impact of the category ″Most used″ on the average time needed to record FoBaST items can only be fully effective with longer-term use. For further optimization of the FoBaST of DNDv2 by means of path analyses, a longer-term study would also be advantageous. The more data are available, the better the FoBaST can be adapted to the target group. However, it should be taken into account that the setting of further studies or data acquisitions plays a major role, since the possible input of the FoBaST depends on the offer of the institution in which the participants are located. Overfitting to a single setting should be avoided. This is especially the case if the resulting version of the DND is meant to be used beyond the period of rehabilitation, as it can be assumed that consumption behavior differs significantly between, e.g., a geriatric rehabilitation division and a home setting. Moreover, maintaining a high level of usability is an ongoing task, as the needs of nutritionists and geriatric patients may change over time due to continuously changing technology commitment and ongoing scientific research. However, the results of the usability of the activity tracker also showed that it is possible to achieve very good results regarding usability even for the target group of geriatric patients.

### Outlook

The data collected using DNDv2, once digitally available, can be used for other use cases with manageable effort, e.g., to use data from DNDv2 and the AT to update data initially collected on the overall nutritional status (MNA or MNA-SF) of a geriatric patient. Furthermore, the data of DNDv2 could be further processed and integrated into existing patient documentation in a similar and fast, visually accessible way, as is the case with the PDS. It would also be useful to link DNDv2 directly to expert software such as OVIVA, especially if the need for nutrition counseling extends beyond a patient’s stay in a geriatric rehabilitation division.

In addition to the further processing and better linking of existing data, the recording process can also be further simplified. One possibility is to integrate an input method assisted by artificial intelligence. To be precise, methods from the field of object detection can further simplify the recording of consumption behavior, but must be adapted to the DNDv2 architecture. The extension of DNDv2 by such technology must still meet the requirements of nutritionists and geriatric patients, otherwise new features could negatively effect the usability of the diary. Consequently, after a successful and automatic classification of FoBaST items, the integration of a method for automatic or assisted quantification of foods, beverages, and dietary supplements would be a consecutive logical step [59].

Moreover, the acquisition of additional data relevant to nutrition counseling should also be considered, e.g., the assessment or classification of stool forms. The form of stool can indicate whether a patient is affected by diarrhea or constipation. It is therefore an important source of information for nutritionists, since such issues can affect the optimal amount or composition of foods, beverages, and dietary supplements [60,61,62]. However, the topic of stool forms may lead to embarrassment and is often avoided or answered only superficially by geriatric patients. The photo-based method, where patients document their stool forms themselves, also has several disadvantages because stools can be obscured by the shape of the toilet or the toilet paper used. In addition, it must be taken into account that geriatric patients are usually in a physically and cognitively impaired state. Accordingly, active self-managed documentation can become a burden. For this reason, research is currently being conducted on an automatic and ambient classification of stool forms to extend the system described in this study [63].

## 5. Conclusions

This study demonstrated that geriatric patients at high risk of frailty syndrome are able, from a usability perspective, to independently log their consumption behavior with the help of a digital nutrition diary (DNDv2). Furthermore, this study showed that commercially available sensor technologies exist that can be used by most geriatric patients, with the exception of the evaluated body composition scale, which could provide DNDv2 with additional data in the future.

Although it is difficult to involve geriatric patients in the development process for any length of time, developers can work with the appropriate use case experts, in this case nutritionists, to develop advanced prototypes that minimize the burden on geriatric patients during the evaluation. Especially with DNDv2, it was shown that the input method based on pictographs and text in combination with an indication of quantities in common household quantities, as well as the overall lean architecture led to high usability. However, it also became clear that geriatric patients need to be continuously involved in the evaluation cycle to accurately tailor usability to their needs. In particular, the evaluation of the usability of the body composition scale showed that even small details can result in usability requirements not being met. Overall, DNDv2 is a good starting point for bringing digitalization to geriatric patients, enabling them to independently log their consumption behavior and, in the future, improving data quantity and data quality for nutritionists. Such an improvement in data could also have a major impact on the nutrition counseling process. With data available online, nutritionists will be able to continuously assess the progress of a therapeutic diet and respond accordingly in a timely manner. Instead of fixed appointments between nutritionists and patients, appointments could be scheduled on an as-needed basis.

## Data Availability

All relevant data is listed in the manuscript. Data are available on reasonable request to the corresponding author.
